# The LPA_3_ Receptor: Regulation and Activation of Signaling Pathways

**DOI:** 10.3390/ijms22136704

**Published:** 2021-06-23

**Authors:** Karina Helivier Solís, M. Teresa Romero-Ávila, Alejandro Guzmán-Silva, J. Adolfo García-Sáinz

**Affiliations:** Departamento de Biología Celular y Desarrollo, Instituto de Fisiología Celular, Universidad Nacional Autónoma de México, Ap. Postal 70-248, Ciudad de México CP 04510, Mexico; samsonyte09@gmail.com (K.H.S.); tromero@ifc.unam.mx (M.T.R.-Á.); aleckz.guzman@gmail.com (A.G.-S.)

**Keywords:** lysophosphatidic acid 3 receptor, LPA_3_, receptor phosphorylation, GRK, PKC, lysophosphatidic acid

## Abstract

The lysophosphatidic acid 3 receptor (LPA_3_) participates in different physiological actions and in the pathogenesis of many diseases through the activation of different signal pathways. Knowledge of the regulation of the function of the LPA_3_ receptor is a crucial element for defining its roles in health and disease. This review describes what is known about the signaling pathways activated in terms of its various actions. Next, we review knowledge on the structure of the LPA_3_ receptor, the domains found, and the roles that the latter might play in ligand recognition, signaling, and cellular localization. Currently, there is some information on the action of LPA_3_ in different cells and whole organisms, but very little is known about the regulation of its function. Areas in which there is a gap in our knowledge are indicated in order to further stimulate experimental work on this receptor and on other members of the LPA receptor family. We are convinced that knowledge on how this receptor is activated, the signaling pathways employed and how the receptor internalization and desensitization are controlled will help design new therapeutic interventions for treating diseases in which the LPA_3_ receptor is implicated.

## 1. Introduction

**Figure 1:**
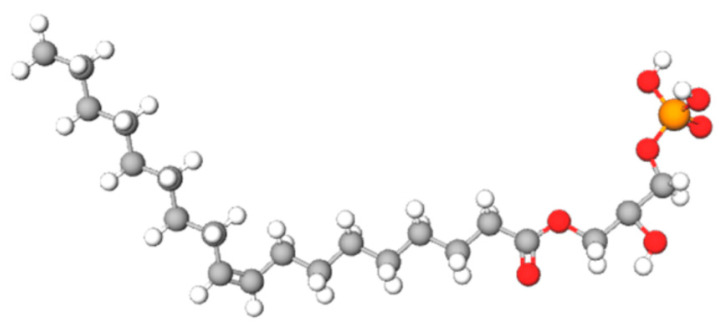
LPA structure. Chemical structure of 1-oleoyl-2-hydroxy-sn-glycerol-3-phosphate (LPA 18:1). Atoms in the chemical structure: Carbon (grey), Hydrogen (white), Oxygen (red), and Phosphorus (orange) (https://pubchem.ncbi.nlm.nih.gov/compound/Lysophosphatidic-acid) (https://molview.org). Accessed on 4 June 2021.

Lysophosphatidic acid (LPA) is a simple lipid comprising a phosphate group and a fatty acid, joined by ester bonds to a glycerol moiety, which is considered the backbone of this molecule [1,2] (Figure 1). LPA has a wide distribution in the organism. It is found in tissues and fluids, likely due to its chemical and physical characteristics, particularly its low molecular weight and water solubility [3].

Two pathways synthesize LPA. In the intracellular pathway, phospholipids (phosphatidylcholine, phosphatidylserine, or phosphatidylethanolamine) or diacylglycerol are the metabolic precursors of LPA through the action of phospholipase D or diacylglycerol kinase, respectively. These enzymes promote the synthesis of phosphatidic acid, which is converted into LPA through catalysis by cytoplasmic lysophospholipases A1 or A2 [4,5]. Other molecules from which LPA is synthesized include glycerol-3-phosphate and monoacyl-glycerol. In these processes, we find the participation of the enzymes glycerophosphate acyltransferase and monoacyl-glycerol kinase, respectively [3,6,7].

In the extracellular pathway, LPA is generated from lysophosphatidylcholine, which is found in the extracellular leaflet of plasma membranes or bound to proteins (such as albumin). In this case, secreted lysophospholipases A1 or A2 split a fatty acid from phosphatidylcholine, synthesizing lysophosphatidylcholine, and then converting it into LPA by a phospholipase D, generally denominated Autotaxin [6,8,9].

LPA is degraded by various enzymes, including LPA acyltransferase, which transfers an acyl group from acyl-CoA to LPA, generating phosphatidic acid; LPA lipid phosphatase, which can remove the phosphate group from LPA, generating monoacylglycerol, and lysophospholipases, which lead to the hydrolysis of the acyl group of LPA, producing a free fatty acid and glycerol 3-phosphate [1,2].

LPA is considered a “bioactive lipid”, implying that it, in addition to its role in phospholipid metabolism, regulates a diverse range of cellular and organism responses such as angiogenesis [8,10,11], neuritic retraction [12,13,14], cell migration [15,16], cell proliferation [17,18,19], reorganization of the cytoskeleton [10,20], development of the central nervous system [8,20,21], neuronal myelination [20,22], pain [23,24], obesity [25], and cancer [26,27,28,29], among many others. These functions are performed by LPA through the activation of six receptors [5,30,31,32,33,34,35].

These receptors are called lysophosphatidic or LPA receptors and are classified into two families. The first family is the lysophospholipid family of receptors, related to those for other phospholipids and including the LPA_1_, LPA_2_, and LPA_3_ receptors. The second family is phylogenetically related to the purinergic receptors and includes the LPA_4_, LPA_5_, and LPA_6_ receptors [1,3,8,36].

**Figure 2:**
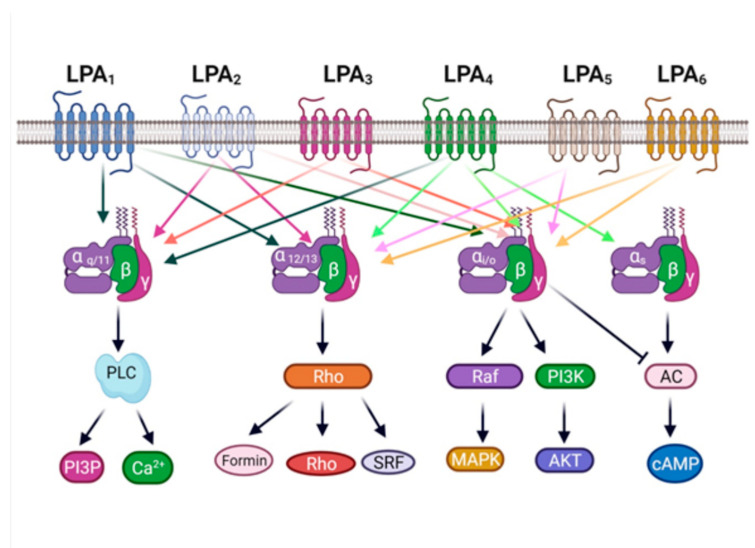
LPA receptors and G proteins. LPA receptors couple with different G proteins that activate distinct signaling pathways. PLC, phospholipase C; PI3K. phosphoinositide 3-kinase; AC, adenylyl cyclase. Created with BioRender.com.

These LPA receptors belong to the G protein-coupled receptor (GPCR) superfamily. They are structurally constituted of seven transmembrane hydrophobic domains connected by three intracellular loops and three extracellular loops, with an extracellular amino-terminal group and an intracellular carboxyl terminus. According to the classification criteria in the GRAFS (Glutamate, Rhodopsin, Adhesion, Frizzled/Taste2, and Secretin groups) system [37] and in the AF system, all of these receptors belong to family A [37,38,39]. These receptors are associated for their signaling with heterotrimeric GTPases or “G” proteins. LPA receptors can activate different Gα proteins (Gα_q/11_ Gα_i/o_, Gα_12/13_, Gα_s_); some of these receptors are considered promiscuous because they can activate different G proteins and downstream signaling pathways that regulate various physiological functions as well as being involved in the pathogenesis of different diseases [5,8,39] (Figure 2).

**Figure 3:**
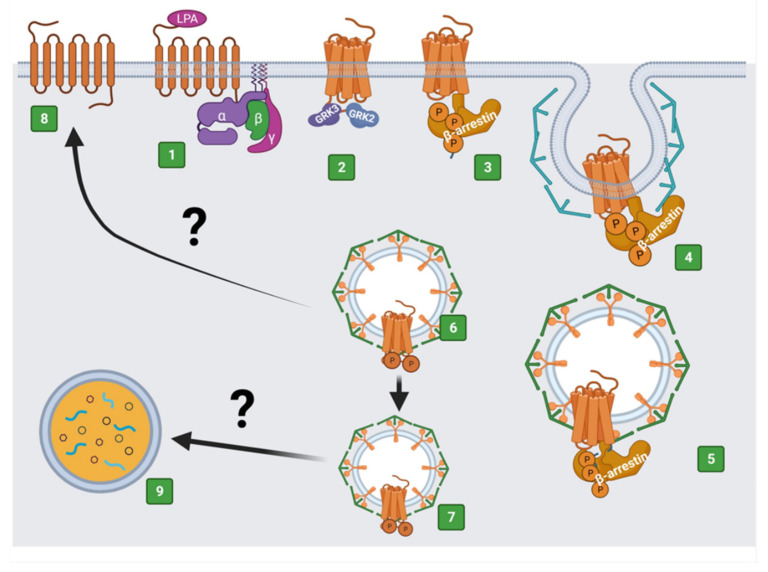
Internalization of agonist-activated LPA_3_ receptors. (1) Activation of LPA_3_ with LPA and recruitment of a G protein. (2) Exposure of GPCR phosphorylation sites. (3) Recruitment of β-arrestin through interaction with phosphorylated sites. (4) Recruitment of the endocytic machinery that initiates receptor endocytosis. (5) Endocytosis of LPA_3_ via endosomes. (6) Receptor-endosomal traffic to (7) lysosomal receptor degradation or (8) receptor recycling to the plasma membrane. Question marks indicate that there is little information on these processes, which are postulated in similarity to what has been defined for other receptors. Created with BioRender.

The activation of GPCRs by their agonists leads to conformational changes promoting heterotrimeric G protein interaction and the exchange of GDP for GTP in their Gα subunits, favoring the dissociation of these heterotrimeric proteins into their Gα subunits, and the βγ complexes, which separately mediate the activation of downstream proteins [8,40,41]. The termination/attenuation of signaling is associated with receptor phosphorylation by different protein kinases (including G protein-coupled receptor kinases (GRKs) and second messenger-activated kinases, among others) [42,43,44,45,46,47,48,49,50]. Such phosphorylations facilitate interaction with β-arrestins, disfavoring receptor-G protein interaction (therefore, decreasing G protein-mediated signaling), recruiting the endocytic machinery, promoting receptor internalization (Figure 3), and activating alternative signaling processes [49,50,51,52,53].

**Figure 4:**
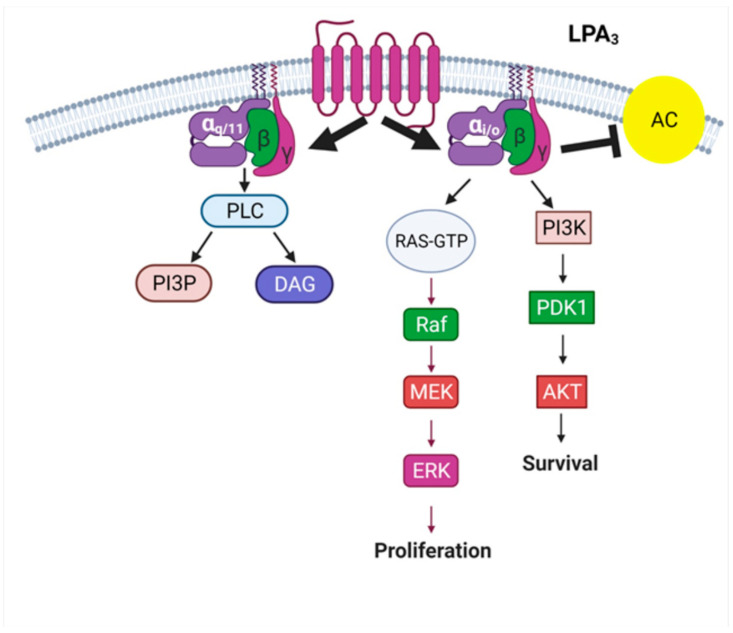
Signaling pathway of LPA_3_ receptors. Activation of this receptor subtype with LPA promotes conformational changes favoring intense interaction with G_αq/11_ and G_αi/o,_ which lead to activation of downstream signaling molecular entities. Abbreviations as in Figure 2. Created with BioRender.

As indicated, the LPA receptors belonging to the lysophospholipid family include the LPA_1-3_ receptors. These receptors have been studied in more detail (reviewed in [8,9]). The LPA_1_ receptor is a 364 amino acid protein, which interacts mainly with G_i/o_, G_q/11_, and G_12/13_. In mice, knocking out the expression of this receptor subtype markedly affects the development of the central nervous system and decreases survival (50% perinatal death). Alteration of LPA_1_ expression has been associated with cancer, neuropathic pain and fibrosis of the lungs. LPA_2_ is a protein of 348 amino acid residues that interacts with G_i/o_, G_q/11_, and G_12/13_. Constitutive receptor loss in mice produces an essentially normal phenotype; however, this receptor contributes to the development and function of synapsis in embryos and adult mice. It has also been associated with some types of cancer and lung functional alterations, such as asthma. The LPA_3_ receptor is a GPCR whose activation mainly promotes the recruitment of two G proteins: Gα_q/11_ and Gα_i/o_; therefore, it is considered promiscuous. The LPA_3_ receptor regulates different signal pathways, as depicted in Figure 4. It should be mentioned that LPA receptors (LPA_1-3_ form homo- and hetero-dimers within the subgroup and hetero-dimers with other receptors such as those of the sphingosine 1-phosphate receptor (S1P_1–3_) and the proton-sensing GPCR, GPR4 [54]. This adds a new level of complexity in signaling and regulation, which we consider important to mention, but it is not considered in the present review.

This review focuses on lysophosphatidic acid receptor 3 (LPA_3_), which participates in different functions which will be briefly described and discussed, and on the information available on the structure of this receptor and its regulation.

## 2. The LPA_3_ Receptor Is Involved in Physiology and Pathology

### 2.1. Antioxidant Enzymes Are Regulated by LPA_3_

LPA_3_ activation appears to play a critical role in regulating the expression of the enzymes that eliminate reactive oxygen species (ROS), primarily through transcription factor NF-E-p45-related factor 2 (Nrf2). Activation of this factor promotes the expression of enzymes such as superoxide dismutase (SOD), glutathione peroxidase 1 (GPx1), heme oxygenase-1 (HO-1), and NAD(P)H: quinone acceptor oxidoreductase (NQO1) [14,55,56]. These enzymes protect cells from the damage produced by the inflammatory process at the beginning of many diseases.

Chen and coworkers [55] showed that LPA_3_ activation increases the expression of antioxidant enzymes in a model of progeria syndrome. In this study, the authors used progerin-transfected HEK293 cells and demonstrated that LPA_3_ activation increases the expression of antioxidant enzymes such as SOD and GPx1, decreasing the damage produced by ROS. Additionally, these authors utilized siRNA to block LPA_3_ expression in mouse fibroblasts, and such treatment reduced the expression of these enzymes, increased ROS production, and induced cellular senescence [55].

These findings are particularly interesting because they suggest that LPA_3_ could be a target to treat diseases in which the participation of ROS promotes cell death. Evidence suggests that in Parkinson’s disease, Alzheimer’s disease, Huntington’s disease, and other degenerative pathologies, increased ROS production is associated with cell death [56,57]. Studies employing a mouse Huntington’s model showed that the stimulation of LPA_1_ and LPA_3_ receptors by gintonin (a complex of LPA molecules and ginseng proteins such as Ginseng major latex-like protein 151) activates the Nrf2 pathway, which protects striatal neurons; the LPA_1/3_ inhibitor, Ki16425, blocked such protection [14].

However, the activation of the LPA_3_ receptors does not always induce a favorable outcome. It has been shown that the Nrf2 transcription factor can promote resistance to chemotherapy [58,59,60,61]. In HL60-DR cells, drug resistance was related to proliferation and a decrease in apoptosis; both events appearing to be related to the activation of the PI3K/AKT (protein kinase also named protein kinase B (PKB))/Nrf2 signal pathway [58,62]. It is interesting to mention that these authors propose a role for LPA_3_/Nrf2/antioxidant enzymes in these actions [62,63]. LPA_3_ receptors activated by their ligands might promote the activation of the Gα_i/o_ protein, which activates PI3K, AKT, and Nrf2, translocation to the nucleus, regulating the transcription of antioxidant enzymes that might promote survival and proliferation.

### 2.2. LPA_3_ Actions in Cardiac Cells and Function

The double-blade (favorable/unfavorable) pattern of action of LPA_3_ has also been observed in the heart. It has been revealed that LPA_3_ activity participates in the survival of cardiomyocytes from neonatal rats during hypoxia by inhibiting the autophagy that occurs in this event [64,65]. In contrast, it was reported that LPA action induced cardiac dysfunction after myocardial infarction in a murine model. These processes could involve the regulation of the PI3K, Rho (Ras homologues, family of small GTPases), and AKT pathways, and the involvement of Gα_i_ and Gα_12/13_ has been proposed [34,64,65,66,67].

Some studies have reported that the activation of LPA_3_ and β-adrenergic receptors promote cardiac hypertrophy; however, the signaling pathways involved appear to be different, at least in H9C2 cardiomyocytes [34]. When LPA_3_ expression is inhibited, an increase in the expression of atrial natriuretic peptide is produced, which inhibits cardiac dysfunction, after myocardial infarction [34]. Additional studies showed that LPA promotes cardiac hypertrophy in cardiomyocytes from neonatal rats and increases cellular apoptosis, cellular elongation, and actin fiber reorganization; it has been suggested that two signaling pathways are involved herein, one through PI3K/AKT/mTOR (protein kinase, mammalian Target Of Rapamycin)/ERK (Extracellular-Signal-Regulated Kinase) 1/2 and the other including NFκB [34,65,66].

### 2.3. Participation of LPA_3_ in Fertility, Embryo Implantation, and Development

During pregnancy, progesterone markedly increases in the blood, and many studies have shown that this hormone increases the expression of LPA_3_ mRNA. This LPA receptor participates in decidualization, implantation, oocyte maturation, and oviduct transport in mice and pigs [68,69]. LPA_3_ expression increases during the first stage of pregnancy, inducing decidualization related to the development of the placenta and the embryo. The importance of LPA_3_ has been evidenced by demonstrating that its knockdown decreases preimplantation with a loss of embryonic spacing, producing the implantation of 3–4 embryos in the same place. This rendered the reabsorption of many embryos, and those that managed to survive were small in size and weight [68,69,70,71]. In males, LPA_3_ and LPA_2_ are expressed in the seminiferous tubules, spermatogonia, and spermatocytes, and such expression is related to male fertility [70].

### 2.4. LPA_3_ in Cancer

Cancer is another pathology in which the expression and action of LPA_3_ receptors are usually associated with a poor prognosis. Studies on breast cancer have shown that LPA_3_ expression was related to metastasis and proliferation in cancer cells [27,72,73]. Several studies have reported that LPA_3_ is expressed in breast cancer tumors (grades 2 and 3), but the signaling pathway by which the receptor regulates this event is not yet fully known [27,72,74]. In addition, there is evidence that LPA_3_ receptors increase proliferation and migration in OVCAR-3 and SKOV3 cells and that these cells lose sensitivity to ultraviolet light and increase their survival rate [19,74,75,76].

Interestingly, LPA_3_ produces drug resistance in pancreatic and hepatic cancer cells. In pancreatic cancer, LPA_3_ expression is considered an indicator or marker of aggressiveness, including metastasis and accelerated regrowth [74,75]. In human sarcoma cells, and in pancreatic and hepatic cancer cells, LPA_3_ activation increases migration, invasion, and metastasis [28,77,78]. These processes are probably related to the increase in matrix metalloproteinases 9 expression, whose proteolytic activity produces the degradation of the extracellular matrix, favoring metastasis. In addition, in hepatoma cells, R777AB, LPA_3_ action is associated with the expression of genes related to drug resistance, such as Mdr (Multidrug resistance protein) 1a and 1b and Gstp1 (Glutathion-S-transferase pi 1) [15,77,78,79,80,81]. The signaling pathways that promote these processes appear to be related the action of Gα_i/o_ and Gα_q_, because the inhibitors of these signaling pathways decrease the proliferation and expression of genes associated with drug resistance and the migration of these cancer cells [79].

It is noteworthy that the increased expression of the LPA_3_ receptors is related to a grim prognosis in cancer; however, this dark side might represent a portal to optimism, if the possibility of LPA_3_ becoming a target for therapeutic intervention is considered.

### 2.5. Other Processes in Which LPA_3_ Participates

LPA_3_ is a receptor that participates during neuritic ramifications after birth. This process is essential because it creates neural networks and maintains information fluxes within neuronal circuits. It has been reported that LPA_3_ activation increases neurite branching in the hippocampus and brain cortex of mice [82,83].

LPA_3_ appears to participate in the maturation of dendritic cells, which are specialized in the immune response. Antagonists and the genetic inhibition of LPA_3_ expression decrease chemotaxis in immature dendritic cells. The process could affect the maturation of these cells, compromising the immune response [32,84]. The mechanisms through which LPA_3_ regulates this process are currently unknown.

LPA_3_ activation also participates in inflammatory processes. LPA_3_ activation promotes the infiltration of interleukins such as IL-6, IL-16, and IL-8 and the synthesis of prostaglandin E2 in patients with rheumatoid arthritis and in fibroblast-like synoviocytes from patients with this disease [85,86]. The signaling pathway through which the receptor produced these events appears to involve Gα_i/o_, which activates the MAPK signaling cascade.

## 3. The LPA_3_ Receptor: Structure and Function

The human LPA_3_ receptor (https://www.uniprot.org/uniprot/Q9UBY5; Accessed on 12 May 2021) is constituted of 353 amino acids (mouse and rat orthologs, 354 amino acids), and its calculated molecular weight is ≈40 KDa (39,998 Da) [5,87,88]. As previously indicated, according to the classification systems GRAFS and A-F, this receptor belongs to the A family [37,38]. LPA_3_ is mainly coupled to two G proteins, Gα_q/11_ and Gα_i/o_; therefore, the G protein-binding motif of this receptor subtype is considered promiscuous. This property allows this receptor to activate different signal pathways, which might explain why it does participate in a large variety of physiological functions and, as previously mentioned, in the pathogenesis of diseases [5,8,89].

**Figure 5:**
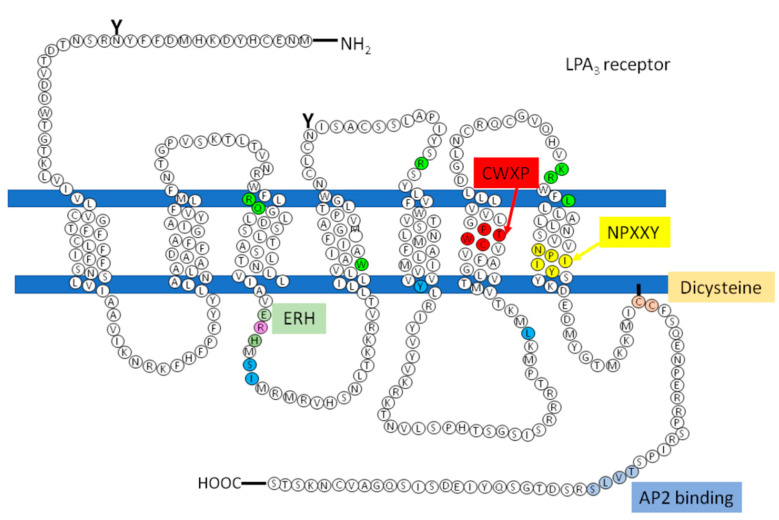
LPA_3_ receptor structure, domains and sites that regulate this receptor. Image shows the amino acid sequence and the organization of the LPA_3_ receptor with three extracellular loops, three intracellular loops, the seven transmembrane domains, the extracellular amino terminus (-NH_2_), and the intracellular carboxyl terminus (-COOH). Colored boxes indicate conserved motifs putatively relevant for activation and regulation of the LPA_3_ receptor. Putative sites where LPA interacts with LPA_3_ are shown in green, while proposed places where GPCRs could be recruiting G proteins are marked in blue and purple (R, arginine that is also part of the ERH motif). “Y” indicates a potential glycosylation site, and the line joining one of the cysteines to the membrane is a putative palmitoylation site.

As a member of the GPCR superfamily, the LPA_3_ receptor is constituted of seven hydrophobic transmembrane domains (TM), which are joined together through three extracellular and three intracellular loops (Figure 5). It is worth mentioning that transmembrane regions are essential for this receptor, as has been observed for others that also belong to the A family. These regions or domains are frequently conserved [90]. For clarity, the sequence indicating the transmembrane domains is presented as (Supplementary Figure S1).

Available information on LPA_3_ receptor structure/function is scarce. Therefore, in order to obtain some information, we performed *in silico* analyses. This allowed us to identify different domains observed in other GPCRs. Among these are the following: an ERH (Glutamic acid-Arginine-Intrahelical hydrogen bonding residue) domain (analogous to the DRY (Aspartic acid-Arginine-Tyrosine) motif) in the transition between the end of TM3 and the initiation of ICL2, a CWXP domain within TM6, an NPXXY domain near the end of TM7, and a di-cysteine domain within the carboxyl terminus (Figure 5). Studies on these domains in other receptors have shown that they are important for the activation and regulation of the GPCRs receptors of the A family [91,92,93,94]. Additionally, an AP2-binding domain is present in the carboxyl terminus [94,95,96].

It is noteworthy to mention that the mutation of these domains usually reduces or abolishes agonist-activation of GPCRs. Studies employing molecular docking showed that ligand binding at GPCRs produced the packaging of TM3-5-6-7 domains; this event was promoted by destabilization of an ionic interaction [92,97], initiating a displacement of TM7 toward TM3 and promoting activation involving the tyrosine residue present in the DRY motif, which is associated with the rotation of the cytoplasmic extreme of TM6 and which promotes the activation of these receptors [92,98,99,100,101].

Additionally, the asparagine residue of the NPXXY motif establishes interactions with other residues, facilitating the movement of TM7 toward TM3 [92,99] and promoting the stability of the activated receptor. Finally, the DRY motif forms a salt bridge with surrounding residues and with TM6; this salt bridge breaks at the moment the ligand binds. The DRY motif creates a new interaction with TM5, stabilizing the receptor in its active conformation, breaking contacts between TM3 and TM6, thus promoting a movement toward the cellular cytoplasm of TM6, which increases the receptor binding to the Gα protein. These events initiate signaling, favor receptor phosphorylation, and later favor association with β-arrestins, all of which are relevant for receptor desensitization [53,92,99,100,101].

The CWXP domain is a motif found in TM6 which seems to participate in the binding of agonists. Rotation of the tryptophan residue causes movements within the binding pocket, promoting the accommodation of the ligand into the receptor. In contrast, the proline residue induces a bend that serves as a pivot for helical movement during receptor activation [92,93,99,101,102]. Other motifs that appear to participate in the activation of GPCRs include the PIF (GPCR microswitch; Proline-Isoleucine-Phenylalanine) motif that is usually found in TM4 and the NPXXY motif found in TM7, both of which are also related to the activation of Gα_q_, Gα_s_, Gα_i_ and β-arrestins [99,103,104,105,106]. It has been shown that in some receptors (such as the histamine 2 receptor [106,107], the formyl peptide receptor [47,100,107], and α- and β-adrenoceptors [93,108], among others), this domain could be regulating agonist-induced internalization, which affects MAPK pathway activation and intracellular calcium mobilization.

The majority of the motifs that generally regulate the activation of GPCRs, including those in the LPA_1_ receptor, have also been found in the LPA_3_ receptor (Figure 5). Only the PIF domain could not be found in the receptor sequence. Therefore, it appears likely that other receptor region(s) could replace the role of PIF in receptor activation.

This illustrates the putative importance of the motifs present in the LPA_3_ receptor at the time of its activation when the ligand binds to it; however, we must recall that the intracellular loops and the carboxyl-terminal region play essential roles, particularly in receptor desensitization and internalization. Current ideas suggest key roles in the phosphorylation of specific residues, mediated by GRKs, second messenger-activated, and other protein kinases [100,109,110].

Other important regions of the LPA_3_ structure are the transmembrane domains, which contain residues that take part in ligand binding. It is worth mentioning that the LPA receptors that belong to the lysophospholipid subfamily entertain an ≈81% similarity among themselves [111,112].

Few studies have reported the participation of these residues during the binding of the ligand in LPA receptors. The residues where LPA has been shown to interact with LPA receptors include arginine 105, glutamine 106, tryptophan 153, arginine 185, lysine 279, and arginine 276 (Figure 5, residues in green). These sites are conserved in the LPA_1_, LPA_2_, and LPA_3_ receptors, but differences appear to exist between these [5,87,89,111,112]. In the case of tryptophan 153, when it was mutated to alanine in the LPA_3_ receptor, it induced a decrease in the potency and efficacy of LPA; such changes were not observed when the LPA_1_ and LPA_2_ receptors were similarly mutated. Likewise, when arginine 279 was substituted with alanine, a decrease in the activation of LPA_1_ and LPA_2_, but not in the LPA_3_ receptor, was observed [111,112].

An amphipathic α-helix is found in the carboxyl terminus of many of the GPCRs of the A family. It is frequently denominated helix 8, and it has a conserved sequence in nearly all of these receptors, i.e., F (R/K) XX (F/L) XXX (L/F); it has been shown that it allows maintaining the surface expression of these GPCRs, promoting GPCR trafficking, and participating in the activation of the G proteins and the receptor’s interaction with the β-arrestins [113,114,115,116].

Zhou and coworkers suggested a mechanism through which many receptors belonging to the GPCR A family could recruit G proteins. It was proposed that in response to agonist-induced conformational changes, residues in transmembrane domains 3, 5, and 6 interact with and activate G proteins [109]. These residues were found in the structure of the LPA_3_ receptor as shown in Figure 5 (indicated in cerulean).

The GRKs are a family of protein kinases that appears to play a major role in the phosphorylation of agonist-occupied GPCRs (Table 1). This family is made up of seven different isoforms that are constituted of a central catalytic domain which is conserved in all GRKs; an amino-terminal area and the carboxyl terminus, both of which differ among these protein kinases, seem to confer them selectivity in their action, and participate in their regulation. These domains constitute the structural basis for their classification into subfamilies; in addition, some GRKs exhibit selective expression in some tissues [117,118,119,120]. The visual GRKs (GRK1 and GRK7) are mainly expressed in the retina, GRK4 is mainly expressed in the testis, whereas the other GRKs (2, 3, 5, and 6) are ubiquitously expressed; visual GRKs have short prenylation sequences (see reviews in [117,120] and references therein). The second subfamily, denominated GRK2 and also, for historical reasons, the β-adrenergic receptor kinase (or βARK) subfamily, exhibits a Pleckstrin homology domain that interacts with G protein βγ dimers and phosphatidylinositol 4, 5-bisphosphate. These kinases are cytoplasmic and their interaction with the plasma membrane seems to occur through these domains. The GRK4 subfamily seems to be bound to the plasma membrane through palmitoylation and/or the presence of positively charged lipid binding elements [117,118,119,120]. It has been proposed that lipids covalently bound to the carboxyl terminus of these proteins, the Pleckstrin homology domain that associates with phosphoinositides, and the polybasic/hydrophobic regions permit these kinases to be recruited to the membrane and to catalyze GPCR phosphorylation at specific residues [119,120,121,122,123].

Such specificity in the GPCR phosphorylation pattern appears to be critical to define subsequent signaling (frequently associated with β-arrestin activation), vesicular trafficking, and the receptor’s fate (rapid or slow recycling to the plasma membrane, or degradation). This has been named the “GPCR phosphorylation barcode,” and numerous research groups are actively working to understand (i.e., to break) this code, which currently is only partially understood [46,50,124,125,126,127,128]. Obviously, initial steps include knowing that the GPCR of interest is actually phosphorylated, the conditions under which that takes place, and the definition of the specific sites affected by such covalent modification. At present, there is evidence that LPA_3_ receptors are phosphorylated in response to agonists and other agents (associated respectively with homologous and heterologous desensitizations) [46,89]. However, to date, the phosphorylation pattern(s) of this receptor is (are) unknown, which seems to be an important gap in our knowledge.

Studies conducted *in silico* showed that the LPA_3_ receptor can be phosphorylated by different protein kinases [89]. Not surprisingly, different isoforms of GRK and PKC are predicted to be responsible for many such phosphorylations; however, other protein kinases such as PKA, PKB/AKT, and some protein tyrosine kinases were present in this *in silico* analysis [129]. Many of these predicted phosphorylation sites could be targeted by several protein kinases [89,129].

**Figure 6:**
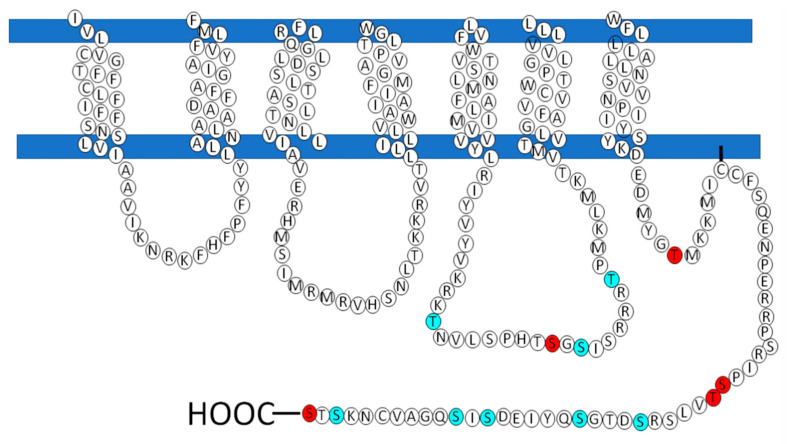
*In silico* prediction of serine and threonine sites phosphorylated by GRK, PKA and PKC. LPA_3_ structure is represented, showing (in red) the putative sites targeted by GRK and (in cerulean) putative sites phosphorylated by PKA or PKC.

Considering the vital role that GRKs play in homologous desensitization/phosphorylation, the putative sites for the action of this family of kinases on LPA_3_ receptor phosphorylation are presented in Figure 6. These residues were obtained in a new analysis employing different and/or updated software programs, including GPS5 (http://gps.biocuckoo.cn; Accessed on 3 April 2021), netphorest (http://netphorest.info; Accessed on 3 April 2021), quokka (https://quokka.erc; Accessed on 4 April 2021) and NetPhos 3.1 (http://www.cbs.dtu.dk; Accessed on 4 April 2021). The criterion used to carry out each study was a high threshold. Only residues that were putative targets of GRK, PKA, or PKC and that obtained a high score were considered. Subsequently, we carried out an analysis on the results obtained and chose the residues that were consistently observed in these analyses; these are presented in Figure 6. The majority of the GRK putative phosphorylation-target residues were found in intracellular loop 3 and the carboxyl terminus region. Not surprisingly, the different software programs used suggested roles of isoforms of the GRK2 and GRK4 subfamilies (Table 1).

The possibility that different GRK isoforms may participate in LPA_3_ phosphorylation is provocative. It has been proposed that GRK 2 and 3 promote receptor endocytosis by the β-arrestin/clathrin pathway more efficiently than other isoforms. At the same time, GRK 5 and 6 appear to mediate β-arrestin-triggered ERK 1/2 signaling [78,117,119,125,130,131,132,133,134]. It is important to mention that GRKs, in addition to carrying out GPCR phosphorylation, can phosphorylate other proteins in the cell cytoplasm that are involved in cell signaling, as well as receptor trafficking proteins such as Gαq and Gβγ, PI3K, clathrin, caveolin, MEK, and AKT/PKB, among others [123,124,135,136,137,138,139,140].

It is noteworthy that the *in silico* analysis suggested that PKA and PKC could participate in LPA_3_ receptor phosphorylation (Figure 6 and Table 2); this result is of interest because it might indicate the involvement of these protein kinases in the heterologous desensitization of this receptor. It has been reported previously that LPA_1–3_ receptors can be phosphorylated in response to the pharmacological activation of PCK with phorbol myristate acetate [89]. However, to the extent of our knowledge, there is no evidence of PKA-induced LPA_3_ receptor phosphorylation. It should be noted that the *in silico* analysis revealed marked overlapping between GRKs, PKA and PKC, suggesting that some sites could be targeted by these groups of kinases (Table 1 and Table 2).

## 4. The Regulation of LPA_3_ Receptors and Its Possible Impact on Signaling

It is worth considering that LPA_3_ receptor phosphorylation has not, to our knowledge, been studied in detail and that the sites actually phosphorylated in whole cells under different stimuli are presently unknown. Similarly, the LPA_3_ receptor internalization process is essentially unexplored. Therefore, we have to assume that the molecular consequences of phosphorylation in signaling and intracellular location/trafficking are similar to those observed for other receptors. Obviously, such assumptions are plainly hypothetical and require experimental confirmation. The *in silico* analysis of the LPA_3_ receptor structure indicates many putative phosphorylation sites, mainly located in intracellular loop 3 and the carboxyl terminus. It seems, therefore, reasonable to suggest that several patterns of phosphorylation might exist and that these might affect signaling, action outcomes, and vesicular traffic. Thus, their study might contribute to a better understanding of the GPCR phosphorylation bar code [89,129].

It has been shown that agonist activation promotes conformational movement of GPCRs, exposing sites that are phosphorylated by GRKs and revealing that such phosphorylations favor the recruitment of β-arrestins [109,110,122,141]. This process has been associated with the phosphorylation patterns mainly observed at the carboxyl termini of GPCRs of the A and B families. In particular, the TXXS motif appears to be of importance for receptor binding to β-arrestins [110,142,143]. Our *in silico* analysis of the LPA_3_ receptor indicates that a domain of this type is present between threonine 326 and serine 329 (TVLS) at the carboxyl terminus (Figure 6); threonine 326 is a putative phosphorylation site of GRKs (Figure 6). It has been shown that the mutation of equivalent sites decreases the interaction of β-arrestins with other GPCRs [109,110,142].

GPCR binding with β-arrestins promotes the recruitment of proteins such as clathrin, dynamin, Adaptin 2, Eps 15, and Rab5, which belong to the endocytic machinery, promoting receptor internalization and early endosome formation. This is followed by internalization with the clathrin assembly lymphoid myeloid leukemia (CALM) protein, as shown in the Epidermal Growth Factor receptor, the LPA_1_ receptor, and other receptors [51,144].

It is noteworthy that Urs and coworkers [143] demonstrated that the LPA_1_ receptor is internalized through different mechanisms when agonist-activated as compared to when it is in response to PKC pharmacological activation; a dileucine domain is critical for PKC-activation induced receptor internalization. No dileucine motif was detected in the LPA_3_ structure. However, other sites appear to be related to internalization, including the following: YXXφ (φ, hydrophobic amino acid; X, any other amino acid); YXT; YXXL; DEXXXLI or DXXLL; NPXY; GDAY; and DIEXXXLL [145,146,147,148,149,150,151]. The YXT domain and the NPXY domain are present in the LPA_3_ receptor. These motifs might participate in LPA_3_ receptor endocytosis, but this must be verified experimentally.

It has been shown that PDZ (structural domain of 80–90 amino-acids; Post-synaptic density protein 95 (PSD-95), Drosophila disc large tumor suppressor (Dlg1), and Zona occludens 1 (ZO-1)) domains participate in the regulation of GPCRs [144,145,146,147,148,149,150,151,152]. In the case of the LPA_1_ receptor, the PDZ domain binds to the GIPC protein, which regulates the function of the receptor and its trafficking, in that both decreasing the expression of GIPC and mutating the PDZ domain induced deficient receptor internalization, promoting its constant activation, and increasing cell proliferation and migration [153]. This event could be directly related to the metastatic processes with which LPA_1_ is associated. Likewise, LPA_2_ has been shown to present a PDZ domain to which the NHERF-2 and MAGI-3 proteins bind, and that could be involved in tumorigenesis, cell invasion, migration, and inflammation [154,155]. The LPA_3_ receptor does not appear to present any canonical PDZ domain.

At present, much is unknown about the regulation of LPA_3_ receptors, which, although part of the LPA receptor family, structurally present possible differential regulatory sites with the other receptors. We conclude that LPA_3_ remains an enigmatic receptor, about which we are just beginning to learn its functions in health and roles in the pathogenesis of diseases but have very little knowledge on its regulation.

## Figures and Tables

**Table 1:** GRKs that putatively phosphorylate different sites in GPCRs.

Subfamilies	GRKs	Domains of Interest
Visual GRKs	GRK1 and GRK7	Prenylation
GRK2 or βARK GRK4	GRK2 and GRK3 GRK4, GRK5 and GRK6	Pleckstrin homology Palmitoylation, polybasic hydrophobic domains

**Table 2:** *In silico* prediction of PKC and PKA phosphorylated LPA_3_ residues.

Position	Amino Acid	PKC/PKA
130	S	PKA
217	T	PKCα/PKCδ/PKCγ
233	T	PKA/PKCδ/PKCι/PKCζ
243	T	PKCi/PKCζ
321	S	PKA/PKCδ/
325	S	PKA/PKC/PKCε
341	S	PKCε
351	S	PKCε

## Data Availability

Not applicable.

## Supplementary Materials

The following are available online at https://www.mdpi.com/article/10.3390/ijms22136704/s1.
