# Supplementary material for: The LPA3 Receptor: Regulation and Activation of Signaling Pathways
Source: Int J Mol Sci. 2021 Jun 23;22(13):6704. doi: 10.3390/ijms22136704 (PMC8269014; doi:10.3390/ijms22136704)
Supplement: Supplementary file 1 [file ijms-22-06704-s001.zip › ijms-1253132-supplementary.pdf]

International Journal of *Molecular Sciences*

MDPI

# Supplementary Figure S1.

The sequence of the LPA3 receptor is presented below, transmembrane domains are indicated in bold type, underlined, and are marked in colors. FASTA Unipro (>sp|Q9UBY5|LPAR3_HUMAN Lysophosphatidic acid receptor 3 OS=Homo sapiens OX=9606 GN=LPAR3 PE=1 SV=1) https://web.expasy.org/

| | | | | |
|---|---|---|---|---|
| 10 | 20 | 30 | 40 | 50 |
| MNECHYDKHM | DFFYNRSNTD | TVDDWTGTKL | **VIVLCVGTFF** | **CLFIFFSNSL** |
| 60 | 70 | 80 | 90 | 100 |
| **VI**AAVIKNRK | FHFPFYY**LLA** | **NLAAADFFAG** | **IAYVFLMF**NT | GPVSKTLTVN |
| 110 | 120 | 130 | 140 | 150 |
| R**WFLRQGLLD** | **SSLTASLTNL** | **LVIA**VERHMS | IMRMRVHSNL | TKKRVT**LLIL** |
| 160 | 170 | 180 | 190 | 200 |
| **LVWAIAIFMG** | **AVPTLGW**NCL | CNISACSSLA | PIYSRS**YLVF** | **WTVSNLMAFL** |
| 210 | 220 | 230 | 240 | 250 |
| **IMVVVYL**RIY | VYVKRKTNVL | SPHTSGSISR | RRTPMKLMKT | **VMTVLGAFVV** |
| 260 | 270 | 280 | 290 | 300 |
| **CWTPGLVVLL** | **L**DGLNCRQCG | VQHVKR**WFLL** | **LALLNSVVNP** | **IIYSYKD**EDM |
| 310 | 320 | 330 | 340 | 350 |
| YGTMKKMICC | FSQENPERRP | SRIPSTVLSR | SDTGSQYIED | SISQGAVCNK |
| 353 | | | | |
| STS | | | | |
